# Quasi-geodesics in relativistic gravity

**DOI:** 10.1140/epjc/s10052-020-08808-9

**Published:** 2021-01-13

**Authors:** Valerio Faraoni, Geneviève Vachon

**Affiliations:** Department of Physics and Astronomy, Bishop’s University, 2600 College Street, Sherbrooke, Québec J1M 1Z7 Canada

## Abstract

A four-force parallel to the trajectory of a massive particle can always be eliminated by going to an affine parametrization, but the affine parameter is different from the proper time. The main application is to cosmology, in which elements of the cosmic fluid are subject to a pressure gradient parallel to their four-velocities. Natural implementations of parallel four-forces occur when the particle mass changes, in scalar–tensor cosmology, and in cosmic antifriction due to particle production.

## Introduction

In General Relativity (GR), massive test particles follow timelike geodesics and particles subject to (non-gravitational) forces deviate from geodesic trajectories. For a freely falling test particle with constant mass $m>0$, let $u^a$ be the four-tangent to the worldline described by this particle in four-dimensional spacetime, normalized to $u_a u^a=-1$ (we follow the notations and conventions of Ref. [1]). The equation of geodesic curves is

1 $$\begin{aligned} u^b \nabla _b u^a = \alpha (\lambda ) u^a, \end{aligned}$$

where $\lambda$ is a parameter along the trajectory, i.e., the tangent to a geodesic trajectory is parallelly transported. It is always possible to change parametrization and use an affine parameter $\sigma$ instead (see the Appendix A); in this parametrization, the geodesic equation becomes

2 $$\begin{aligned} u^c \nabla _c u^a = \frac{du^b}{d\sigma }+\varGamma ^{a}_{bc} u^b u^c = 0, \end{aligned}$$

where $\varGamma ^a_{bc}$ denotes the Christoffel symbols and now $u^{\mu } = dx^{\mu }/d\sigma$. The right hand side of Eq. (1) can always be removed, as one would expect on the basis of the Equivalence Principle. In fact, no force acts on a geodesic particle and geodesic curves are completely determined by the geometry (i.e., by gravity), which can be made flat locally by going to a freely falling frame; this fact makes one think that all appearances of a gravitational force in this frame, including the right hand side of Eq. (1), are spurious.

Now, all the other possible affine parameters $\sigma '$ are related to $\sigma$ by an affine transformation $\sigma \rightarrow \sigma '= a_0 \sigma +b_0$, where $a_0$ and $b_0$ are constants (e.g., [2]). In GR, it is customary to use the proper time $\tau$ as an affine parameter along timelike geodesics because it is the time measured by a clock carried by the observer freely falling on that geodesic and is, therefore, a privileged parameter from the physical point of view.

A test particle subject to a non-gravitational four-force $F^a$ will follow a trajectory that deviates from a geodesic and obeys the equation

3 $$\begin{aligned} m \, s^c \nabla _c s^a= F^a \end{aligned}$$

(here and in the following, we denote the four-tangent to a geodesic with $u^c$ and that to a non-geodesic trajectory with $s^c$). According to standard terminology, the particle’s four-acceleration $a^b \equiv s^c \nabla _c s^b$ is always orthogonal to the four-velocity $s^c$ in the 4-dimensional sense, $a^c s_c=0$, as follows from covariantly differentiating the normalization relation $s^c s_c=-1$ and then, if the particle mass *m* is constant, $F^c =ma^c$ is also orthogonal to the particle’s trajectory. A force (or a component of the acceleration) tangent to the particle’s four-velocity $s^c$ can always be eliminated by a reparameterization, whether it is of gravitational origin ( i.e., pure geometry showing up because of a non-affine parametrization) or of non-gravitational nature. The procedure to remove the right hand side of Eq. (1) (reported in the Appendix A) does not care whether the tangent force is gravitational or not and applies to all tangent forces. We have in mind a specific situation occurring in cosmology, which will be discussed in Sect. 2. If the force $F^a$ is non-gravitational, the function $\alpha (\lambda )$ and, consequently, the transformation to the geodesic proper time, will depend on the particle mass *m* since the Equivalence Principle is unique to gravity and does not apply to non-gravitational forces.

The trajectories of particles subject to forces *parallel* to the four-tangent to the trajectory are, from the purely mathematical point of view, geodesic curves and can be affinely parametrized. If one begins with the equation

4 $$\begin{aligned} \frac{ds^a}{d\tau _c} +\varGamma ^a_{bc} s^b s^c = \alpha (\tau _c) \, s^a, \end{aligned}$$

where $$\tau _c$$ is the proper time of the particle (i.e., the time measured by a clock at rest with respect to the particle), the affine parameter $$\tau $$ that removes the force will be different from $$\tau _c$$. In other words, the proper time $$\tau _c$$ of the particle subject to a force and the proper time $$\tau $$ of the timelike geodesic obtained from it will differ. Since the four-force $$F^a $$ has no component orthogonal to $$s^a$$ (in the 4-dimensional sense), the tangent $$s^c$$ to the particle trajectory (parametrized by the proper time $$\tau _c$$) can only deviate from the four-tangent $$u^c$$ to the corresponding geodesic in the time component and in the spatial component parallel to $$s^i$$ in the 3-dimensional sense. In a local chart $$\left\{ x^{\mu } \right\} $$, we have

5 $$\begin{aligned}&s^{\mu } \equiv \frac{dx^{\mu }}{d\tau _c} = ( s^0, \mathbf {s} )= \frac{dx^{\mu }}{d\tau }\, \frac{d\tau }{d\tau _c} \equiv \frac{d\tau }{d\tau _c} \, u^{\mu } \equiv \gamma \, u^{\mu } \nonumber \\&\quad =\gamma ( u^0, \mathbf {v} ), \end{aligned}$$

where $\gamma (v) \equiv d\tau /d\tau _c$ is the instantaneous Lorentz factor of the Lorentz boost relating the freely falling observer (which moves with 3-velocity $\mathbf {v}$ in the spatial direction of the trajectory) and the particle subject to the force $F^a$. The 3-spaces perceived by the observers $s^a$ and $u^a$ have Riemannian metrics

6 $$\begin{aligned} h_{ab}= & {} g_{ab} + s_as_b = g_{ab} + \left( \frac{d\tau }{d\tau _c} \right) ^2 u_a u_b \end{aligned}$$

7 $$\begin{aligned} \gamma _{ab}= & {} g_{ab} + u_a u_b, \end{aligned}$$

respectively. In order to eliminate the parallel force from the motion of the particle, one has to abandon its proper time as the parameter along the trajectory and adopt the affine parameter instead, which is equivalent to a Lorentz boost in the spatial direction of the trajectory in 3-space by a Lorentz factor dependent on the position along the trajectory. This means shifting time intervals, lengths, frequencies, and energies with respect to the frame comoving with the particle. Therefore, although from the mathematical point of view there is no difference between a geodesic and the trajectory of a particle subject to a parallel force, from the physical point of view there is a fundamental difference consisting of the fact that the affine parameter eliminating the force is not the proper time which is the physically preferred parameter along the trajectory. In other words, the two worldlines correspond to different physical observers. We propose to call “quasi-geodesics” the timelike trajectories of massive particles subject to a force parallel to the trajectory’s four-tangent, for which the proper time is not an affine parameter.

Several coordinates systems used in GR are based on timelike geodesics (i.e., on freely-falling observers) and are used in the context of black hole physics and horizon thermodynamics. In the Schwarzschild geometry, Painlevé–Gullstrand coordinates [10, 11] are based on radial timelike geodesics. They correspond to the coordinates attached to freely falling observers released from rest from infinity and traveling radially inward. The more general Martel–Poisson family of coordinate systems is obtained when freely falling radial observers are released with non-zero initial velocity [3]. This family of coordinates contains Painlevé–Gullstrand coordinates as a special case and has as a limit the more familiar Eddington–Finkelstein coordinates [4, 5]. Painlevé–Gullstrand and Martel–Poisson coordinates have been generalized to arbitrary static and asymptotically flat black hole spacetimes in [3] and to de Sitter and other static universes in [6, 7]. Other coordinates based on radial timelike geodesics in the Schwarzschild geometry are the Novikov and the Gautreau–Hoffman coordinates, corresponding to observers launched at a finite radius [8, 9].^1^

## Perfect fluids

Let us consider a perfect fluid described by the tensor

8 $$\begin{aligned} T_{ab}=\left( P+\rho \right) s_a s_b +P g_{ab} , \end{aligned}$$

where $g_{ab}$ is the metric tensor, $\rho$ is the energy density, *P* is the pressure, and $s^c$ is the fluid four-velocity (normalized to $s_c s^c=-1$). The covariant conservation equation $\nabla ^b T_{ab}=0$ for this perfect fluid reads

9 $$\begin{aligned}&s_a \left[ s^b \nabla _b \left( P+\rho \right) \right] +\left( P+\rho \right) s^b \nabla _b s_c \nonumber \\&\qquad +\left( P+\rho \right) s_a \nabla ^b s_b +\nabla _a P =0. \end{aligned}$$

Observers comoving with the fluid (“comoving observers”), i.e., with four-velocity $s^a$, perceive the 3-dimensional space as endowed with the metric (6). The mixed tensor ${h^a}_b$ is a projector onto this 3-space, because $h_{ab} s^a=h_{ab}s^b=0$. Also $\gamma _{ab} s^a=\gamma _{ab} s^b= \gamma _{ab} u^a = \gamma _{ab} u^b=0$. By projecting Eq. (9) along the time direction $s^a$ of the comoving observers, one obtains the time component of the covariant conservation equation

10 $$\begin{aligned} \frac{d\rho }{d\tau _c} +\left( P+\rho \right) \nabla ^b u_b=0 , \end{aligned}$$

where $\tau _c$ denotes the proper time of the comoving observers. By projecting Eq. (9) onto the 3-space “seen” by the comoving observers (i.e., by contracting with the projector ${h^a}_b$), one obtains instead

11 $$\begin{aligned} {h^a}_c \nabla _a P + \left( P+\rho \right) h_{cb} a^b =0 , \end{aligned}$$

where $a^b \equiv s^c \nabla _c s^b$ is the particle’s four-acceleration. Let us consider fluid elements, regarded as “fluid particles”. In the absence of external forces, these fluid particles are only subject to gravity and to the pressure gradient $\nabla _a P$. In the case of dust with $P \equiv 0$, the covariant conservation equation $\nabla ^b T_{ab}=\nabla ^b \left( \rho u_a u_b \right) =0$ contains the result that dust particles follow geodesics. In fact, the time component of this equation gives

12 $$\begin{aligned} \frac{d\rho }{d\tau } + \rho \nabla ^b u_b=0 \end{aligned}$$

which, substituted into the spatially projected conservation equation

13 $$\begin{aligned} u_a \left( u^b \nabla _b \rho +\rho \nabla ^b u_b \right) + \rho u^b \, \nabla _b u_a=0 \end{aligned}$$

produces the affinely parametrized geodesic equation $u^b \nabla _b u^a=0$. This is the celebrated “geodesic hypothesis”, i.e. the result that test (or dust) particles follow geodesics. This result is contained in the general formalism of GR, does not necessitate a separate assumption [1], and contains the additional ingredient that the proper time of these test particles is an affine parameter along the geodesics.

The situation is different for a general perfect fluid with pressure. The conservation equation (10) does not contain the pressure gradient, while the spatial equation (11) does. If $\nabla _aP$ has a component along the time direction (of the comoving observers), i.e., along $s^a$, this component is annihilated by projecting onto the 3-space orthogonal to $s^a$ and Eq. (11) will be completely insensitive to it. Therefore, the equations of motion of the fluid particles expressed by $\nabla ^b T_{ab}=0$ are completely insensitive to a component of the pressure gradient parallel to the four-velocity $s^a$. If the pressure gradient $\nabla ^a P$ is exactly parallel to $s^a$, it drops out completely from the equations describing the trajectories of the fluid particles (the pressure *P* itself still plays a role, since it gravitates and curves spacetime together with the energy density $\rho$). Therefore, the particle trajectories may look as if there were no forces (i.e., geodesic trajectories), but there is the important difference that the proper time $\tau _c$ along the particle trajectory does not coincide with the proper time along the corresponding geodesic and the observers $s^c$ and $u^c$ perceive different 3-spaces Lorentz-boosted with respect to each other.

## Cosmology

The distinction between freely falling frame and comoving frame along a quasi-geodesic trajectory becomes essential in cosmology, where the universe is permeated by a perfect fluid, or by a mixture of perfect fluids with the same four-velocity (then the partial densities and the partial pressures simply add up and we can consider a tensor (8) with $\rho$ and *P* equal to the *total* energy density and pressure). The spatial homogeneity and isotropy of the cosmic microwave background and of large scale structures (apart from small perturbations) makes the comoving observers assume a privileged role: they are the observers who see the cosmic microwave background as homogeneous and isotropic around them (apart from tiny temperature perturbations $\delta T/T \sim 5 \times 10^{-5}$) and cosmological observations are usually referred to these observers.

The Friedmann–Lemaître–Robertson–Walker (FLRW) line element in comoving coordinates $\left( t,r,\vartheta , \varphi \right)$ is

14 $$\begin{aligned} ds^2 =-dt^2 +a^2(t) \left( \frac{dr^2}{1-kr^2} +r^2 d\varOmega _{(2)}^2 \right) , \end{aligned}$$

where $d\varOmega _{(2)}^2 =d\vartheta ^2 +\sin ^2 \vartheta \, d\varphi ^2$ is the line element of the unit 2-sphere, *k* is the curvature index, and the comoving time *t* is the proper time of the comoving observers. We assume that the matter source is a perfect fluid described by the tensor (8). The comoving observers coincide with the geodesic observers if and only if this fluid is a dust with $P\equiv 0$.

Let us examine the geodesic equation in the geometry (14). The only non-vanishing Christoffel symbols are

15 $$\begin{aligned} \varGamma ^0_{11}= & {} \frac{a{\dot{a}}}{1-kr^2},\quad \varGamma ^0_{22}=a{\dot{a}}r^2 ,\quad \varGamma ^0_{33}=a{\dot{a}}r^2 \sin ^2 \vartheta , \end{aligned}$$

16 $$\begin{aligned} \varGamma ^1_{01}= & {} \varGamma ^2_{02}= \varGamma ^3_{03} = \frac{{\dot{a}}}{a},\quad \varGamma ^1_{22}= -r ( 1-kr^2), \end{aligned}$$

17 $$\begin{aligned} \varGamma ^1_{33}= & {} -r ( 1-kr^2) \sin ^2 \vartheta ,\quad \varGamma ^2_{33} = -\sin \vartheta \cos \vartheta , \end{aligned}$$

18 $$\begin{aligned} \varGamma ^3_{22}= & {} \cot \vartheta ,\quad \varGamma ^2_{12}=\varGamma ^3_{13}= \frac{1}{r}, \end{aligned}$$

and those related to them by the symmetry $\varGamma ^a_{bc}=\varGamma ^a_{cb}$ and an overdot denotes differentiation with respect to the comoving time *t*. Radial timelike geodesics are parametrized by the proper time $\tau$ of the freely falling observers and have $u^{\vartheta }=d\vartheta /d\tau = u^{\varphi }=d\varphi /d\tau =0$. The radial component of the geodesic equation is

19 $$\begin{aligned} \frac{du^r}{d\tau } + 2H u^t u^r =0, \end{aligned}$$

where $H \equiv {\dot{a}}/a$ is the Hubble function. This equation is immediately integrated to

20 $$\begin{aligned} u^r=u^r_{(0)} \, \frac{a_{(0)}^2}{a^2}, \end{aligned}$$

where $u_{(0)}^r \equiv u^r( \tau _0)$ is the initial condition at a point $\tau _0$ along the geodesic trajectory, where *a* assumes the value $a_0$. The normalization of the four-velocity $g_{ab}u^a u^b=-1$ gives its time component as

21 $$\begin{aligned} u^t =\sqrt{ 1+ \frac{( u^r_{(0)} )^2 a_{(0)}^4 }{a^2 (1-kr^2) } }. \end{aligned}$$

Let the massive particle (or geodesic observer) be initially at rest at the point of comoving coordinates $\left( t_0, r_0, \vartheta , \varphi \right)$, or

22 $$\begin{aligned} u^{\mu }_{(0)} =\left( 1, 0,0,0 \right) . \end{aligned}$$

Then, the timelike radial geodesic with this initial condition has tangent $u^{\mu } =\left( 1, 0,0,0 \right)$ at all subsequent times. In particular, $u^t\equiv dt/d\tau =1$ and comoving and proper time coincide (apart from a possible shift in the origin). This is not true for any radial timelike geodesics, but only for those satisfying the special initial condition $u^a_{(0)}= s^a_{(0)}$. In other words, particles can move outward radially at any speed describing the same spacetime trajectories, but if their radial velocities are synchronized initially with the Hubble flow, they remain synchronized and their proper time then coincides with the comoving time.

We can now find the relation between the proper times *t* and $\tau$ of comoving and freely falling (i.e., geodesic) observers. The time component of the geodesic equation is

23 $$\begin{aligned} \frac{du^t}{d\tau } + \frac{a_{(0)}^4 {\dot{a}}}{a^3( 1-kr^2) } ( u_{(0)}^r )^2 =0 \end{aligned}$$

and the initial condition $u_{(0)}^r=0$ yields

24 $$\begin{aligned} t(\tau ) = C_1\tau + C_2, \end{aligned}$$

where the $C_{i}$ are integration constants. If the special radial timelike geodesics satisfy the initial condition $u^a_{(0)}= s^a_{(0)}$, it is also $C_1=1$ and not only the set of spacetime points lying along the geodesic curve and the comoving observer’s worldline coincide, but also their parametrizations coincide. Thus, if freely falling observers are given a special initial velocity that synchronizes them with the Hubble flow initially, they remain in the Hubble flow at all subsequent times. Comoving time and the proper time of freely falling observers then coincide, but it is important to realize that this does not happen for all radial timelike geodesics, only for those that satisfy the special (synchronizing) initial condition.

## Physical nature of a parallel force

### A parallel force cannot be electromagnetic

A four-force parallel to the particle trajectory cannot be an electromagnetic force. In fact, the electromagnetic four-force on a test charge *q* is

25 $$\begin{aligned} F^{a} = qF^{ab}s_{b}, \end{aligned}$$

where $s^a$ is the four-tangent to the particle worldline and $F_{ab}$ is the Maxwell tensor. In the frame of this particle, the components of the four-tangent are $s^{\mu }=\left( s^0,0,0,0\right)$ and $F^{\mu }=q F^{\mu 0}s_0$ is purely spatial because, for $\mu =i=1,2,3$, $F_{0i}=- E_i$, where $E^i$ is the electric field perceived by the particle. For $\mu =0$, one has $F^{00}=0$ due to the antisymmetry of the Maxwell tensor, and the electric force cannot have a time component and must be purely spatial. Similarly, the magnetic force cannot have a time component because the purely spatial magnetic field $B^{\mu }$ is built out of the space-space component $F_{ij}$ of $F_{\mu \nu }$ according to

26 $$\begin{aligned} F_{\mu \nu }=\left( \begin{array}{cccc} 0 &{} -E_x &{} -E_y &{} -E_z \\ E_x &{} 0 &{} B_z &{} -B_y \\ E_y &{} -B_z &{} 0 &{} B_x\\ E_z &{} -B_y &{} B_z &{} 0 \\ \end{array} \right) \end{aligned}$$

in local Cartesian coordinates (this is even more intuitive, since the Lorenz force due to a purely magnetic field is perpendicular to the particle’s 3-velocity in the 3D sense). Therefore, a force parallel to the worldline of a massive particle cannot be of electromagnetic nature.

### Parallel force due to a variable particle mass

A four-force parallel to the trajectory (with four-tangent $s^a$) is akin to a variable particle mass. In fact, the 4-dimensional analogue of Newton’s second law

27 $$\begin{aligned} F^a =\frac{Dp^a}{D\tau } = \frac{ D(m s^a)}{D\tau } = \frac{dm}{d\tau } \, s^a + m \, \frac{Ds^a}{D\tau }, \end{aligned}$$

where $\tau$ is the proper time along the trajectory, can be rewritten as

28 $$\begin{aligned} \frac{Ds^a}{D\tau } \equiv \frac{ds^a}{d\tau } +\varGamma ^a_{bc}s^b s^c =-\left[ \frac{d }{d\tau } \, \ln \left( \frac{m }{m_0} \right) \right] s^a, \end{aligned}$$

where $m_0$ is a constant with the dimensions of a mass. The variation of the mass $m(\tau )$ along the trajectory generates a force $F^a = - \, \frac{dm}{d\tau } \, s^a$ parallel to it. This force can be eliminated by a reparametrization (as done in the Appendix A), however the affine parameter $\lambda$ that achieves this is different from the proper time $\tau$ measured by the observer. *Vice-versa*, a force parallel to the trajectory of a particle can be interpreted as a variation of the particle’s mass along the trajectory by setting

29 $$\begin{aligned} \frac{d}{d\tau } \ln \left( \frac{m}{m_0} \right) =- \alpha (\tau ) , \end{aligned}$$

which gives the mass dependence

30 $$\begin{aligned} m(\tau ) = m_0 \, \text{ e}^{-\int d\tau \, \alpha (\tau )}. \end{aligned}$$

By using Eq. (A.6), the affine parameter is found to be

31 $$\begin{aligned} \sigma (\tau ) =A \int d\tau \, \frac{m(\tau )}{m_0} +B. \end{aligned}$$

This discussion applies regardless of the physical process causing the variation of the particle mass. Variable masses are encountered, for example, in rockets^2^ and in solar sails (e.g., [37–39]).

The variation of particle masses in cosmology has been studied in Refs. [13–18], mostly in the context of scalar–tensor gravity, which is discussed next.

### Parallel forces in scalar–tensor cosmology

The (Jordan frame) Brans–Dicke action is

32 $$\begin{aligned} S^{(BD)}= & {} \frac{1}{16\pi } \, \int d^4 x \, \sqrt{-g} \nonumber \\&\times \left[ \phi R -\frac{\omega }{\phi } \, g^{cd} \nabla _c\phi \, \nabla _d\phi -V( \phi ) \right] + S^{(m)}, \end{aligned}$$

where

33 $$\begin{aligned} S^{(m)}=\int d^4 x \, \sqrt{-g} \, {{{\mathcal {L}}}}^{(m)} \end{aligned}$$

is the matter action, $\phi >0$ is the Brans–Dicke scalar field, and the dimensionless parameter $\omega$ is the Brans–Dicke coupling. The conformal transformation of the metric

34 $$\begin{aligned} g_{ab}\rightarrow {\tilde{g}}_{ab}=\varOmega ^2 \, g_{ab}, \quad \varOmega =\sqrt{\phi }, \end{aligned}$$

and the scalar field redefinition

35 $$\begin{aligned} {\tilde{\phi }}( \phi )= \sqrt{ \frac{2\omega +3}{16\pi G} } \, \ln \left( \frac{\phi }{\phi _0} \right) \end{aligned}$$

(where $\omega > -3/2$) bring the Brans–Dicke action (32) into its Einstein frame form [19]

36 $$\begin{aligned} S= & {} \int d^4 x \, \left\{ \sqrt{ -{\tilde{g}}} \left[ \frac{ {\tilde{R}}}{16\pi G} -\frac{1}{2} \, {\tilde{g}}^{ab} {\tilde{\nabla }}_a{\tilde{\phi }} {\tilde{\nabla }}_b{\tilde{\phi }} -U\left( {\tilde{\phi }} \right) \right] \right. \nonumber \\&\left. + \text{ e}^{ -8\sqrt{ \frac{\pi G}{2\omega +3} } \,\, {\tilde{\phi }} } {{{\mathcal {L}}}}^{(m)} \left[ {\tilde{g}} \right] \right\} , \end{aligned}$$

where ${\tilde{\nabla }}_a$ is the covariant derivative operator of the rescaled metric ${\tilde{g}}_{ab}$,

37 $$\begin{aligned} U\left( {\tilde{\phi }} \right) = V\left[ \phi \left( {\tilde{\phi }} \right) \right] \exp \left( -8 \sqrt{\frac{\pi G}{2\omega +3} } \, {\tilde{\phi }} \right) \, \end{aligned}$$

and a tilde denotes Einstein frame quantities. (The redefinition (35) has the purpose of casting the scalar field kinetic energy density into canonical form.) In the Einstein frame, the matter Lagrangian density is multiplied by an exponential factor with argument proportional to ${\tilde{\phi }}$ (Eq. (36)): this scalar couples explicitly to matter.

In general, the covariant conservation equation for the matter tensor $\nabla ^{b} \, T_{ab}^{(m)} =0$ is not conformally invariant [1]: the conformally transformed ${\tilde{T}}_{ab}^{(m)}$ satisfies instead

38 $$\begin{aligned} {\tilde{\nabla }}^{b} \, {\tilde{T}}_{ab}^{(m)} =-\,\frac{d}{d\phi } \left[ \ln \varOmega (\phi ) \right] \, {\tilde{T}}^{(m)} \, {\tilde{\nabla }}_a \phi . \end{aligned}$$

Only conformally invariant matter with $$T^{(m)} =0 $$ is conformally invariant.

As done in Sect. 1 for dust, the Einstein frame modification of the geodesic equation can be derived from Eq. (38). Timelike geodesics of the original metric $g_{ab}$ are not mapped into geodesics of ${\tilde{g}}_{ab}$ because of the force proportional to ${\tilde{\nabla }}^a \phi$ introduced by the conformal transformation. When applied to the Einstein frame action, the tensor expression

39 $$\begin{aligned} {\tilde{T}}_{ab}^{(m)}=\frac{-2}{\sqrt{ -{\tilde{g}} } } \, \frac{ \delta \left( \sqrt{-{\tilde{g}}} \, \, {{{\mathcal {L}}}}^{(m)} \right) }{\delta {\tilde{g}}^{ab} }, \end{aligned}$$

yields

40 $$\begin{aligned} {\tilde{T}}_{ab}^{(m)}= & {} \varOmega ^{-2} \, T_{ab}^{(m)}, \;\;\;\; \widetilde{ { {T_{a}}^{b}}^{(m)} } = \varOmega ^{-4} \, {{T_a}^b}^{(m)} , \end{aligned}$$

41 $$\begin{aligned} {{\tilde{T}}}^{ab}= & {} \varOmega ^{-6} \, {T^{ab}}^{(m)}, \end{aligned}$$

and

42 $$\begin{aligned} {\tilde{T}}^{(m)}= \varOmega ^{-4} \, T^{(m)}. \end{aligned}$$

For a perfect fluid with tensor (8), the conformal map generates

43 $$\begin{aligned} {\tilde{T}}_{ab}^{(m)}=\left( {\tilde{P}}^{(m)}+{\tilde{\rho }}^{(m)} \right) {\tilde{u}}_a {\tilde{u}}_b +{\tilde{P}}^{(m)} \, {\tilde{g}}_{ab}, \end{aligned}$$

in the rescaled world, where ${\tilde{g}}_{ab} \, {\tilde{u}}^a {\tilde{u}}^b=-1$, from which one obtains

44 $$\begin{aligned} {\tilde{u}}^a=\varOmega ^{-1} \, u^a,\quad {\tilde{u}}_a=\varOmega \, u_a. \end{aligned}$$

Equations (41), (43), and (44) yield

45 $$\begin{aligned}&( {\tilde{P}}^{(m)} + {\tilde{\rho }}^{(m)}) {\tilde{u}}_a {\tilde{u}}_b + {\tilde{P}}^{(m)} \, {\tilde{g}}_{ab} \nonumber \\&\quad = \varOmega ^{-2}\left[ ( P^{(m)} +\rho ^{(m)} ) u_a u_b + P^{(m)} \, g_{ab} \right] , \end{aligned}$$

from which the transformation properties

46 $$\begin{aligned} {\tilde{\rho }}^{(m)}=\varOmega ^{-4} \, \rho ^{(m)},\quad {\tilde{P}}^{(m)}=\varOmega ^{-4} \, P^{(m)} \end{aligned}$$

follow. If the Jordan frame fluid is described by the equation of state

47 $$\begin{aligned} P^{(m)}=w \rho ^{(m)} \end{aligned}$$

with $w=$constant, the same equation of state is valid in the Einstein frame due to Eq. (46).

In FLRW spacetimes, the Jordan frame fluid conservation equation

48 $$\begin{aligned} \frac{d \rho ^{(m)}}{dt} +3H ( P^{(m)} +\rho ^{(m)} )=0 \end{aligned}$$

is mapped into the Einstein frame equation

49 $$\begin{aligned}&\frac{d {\tilde{\rho }}^{(m)}}{dt}+3 {\tilde{H}} ( {\tilde{P}}^{(m)} +{\tilde{\rho }}^{(m)} )\nonumber \\&\quad =\frac{d \left( \ln \varOmega \right) }{d\phi } \,\, {\dot{\phi }} ( 3{\tilde{P}}^{(m)} - {\tilde{\rho }}^{(m)} ). \end{aligned}$$

Let us return to general spacetimes. Under the conformal rescaling, the tensor $T_{ab}^{(m)}$ scales according to

50 $$\begin{aligned} {\tilde{T}}^{ab}_{(m)}=\varOmega ^s \, \, T^{ab}_{(m)}, \quad {\tilde{T}}_{ab}^{(m)}=\varOmega ^{s+4} \,\, T_{ab}^{(m)}, \end{aligned}$$

where *s* is an appropriate conformal weight and the Jordan frame covariant conservation equation $\nabla ^b \, T_{ab}^{(m)}=0$ maps (in four spacetime dimensions) to [1, 12]

51 $$\begin{aligned} {\tilde{\nabla }}_a \left( \varOmega ^s \, T^{ab}_{(m)} \right)= & {} \varOmega ^s \, \nabla _a T^{ab}_{(m)} +\left( s+6 \right) \varOmega ^{s-1} \, T^{ab}_{(m)}\nabla _a \varOmega \nonumber \\&- \varOmega ^{s-1} g^{ab} \, T^{(m)} \nabla _a \varOmega . \end{aligned}$$

Choosing the conformal weight $s=-6$ yields, consistently with Eq. (42),

52 $$\begin{aligned} {\tilde{T}}^{(m)} \equiv {\tilde{g}}^{ab} \, {\tilde{T}}_{ab}^{(m)}= \varOmega ^{-4} \,\, T^{(m)} \end{aligned}$$

and Eq. (51) is mapped to

53 $$\begin{aligned} {\tilde{\nabla }}_a {\tilde{T}}^{ab}_{(m)} =- {\tilde{T}}^{(m)}\, {\tilde{g}}^{ab} \, {\tilde{\nabla }}_a \left( \ln \varOmega \right) . \end{aligned}$$

For Brans–Dicke theory with $\varOmega =\sqrt{G\phi }$, [12, 20]

54 $$\begin{aligned} {\tilde{\nabla }}_a {\tilde{T}}^{ab}_{(m)} =- \frac{1}{2\phi } \,\, {\tilde{T}}^{(m)} \, {\tilde{\nabla }}^b \phi = - \sqrt{ \frac{4\pi G}{2\omega +3} } \, \, {\tilde{T}}^{(m)} \,\, {\tilde{\nabla }}^b {\tilde{\phi }}, \end{aligned}$$

from which one derives the corrected geodesic equation. For a dust fluid with $P=0$, one obtains

55 $$\begin{aligned}&{\tilde{u}}_a \, {\tilde{u}}_b \, {\tilde{\nabla }}^b {\tilde{\rho }}^{(m)} +{\tilde{\rho }}^{(m)} \, {\tilde{u}}_a \, {\tilde{\nabla }}^b {\tilde{u}}_b +{\tilde{\rho }}^{(m)} \, {\tilde{u}}_c \,{\tilde{\nabla }}^c \, {\tilde{u}}_a \nonumber \\&\quad - \sqrt{ \frac{4\pi G}{2\omega +3} } \, \, {\tilde{\rho }}^{(m)} \, {\tilde{\nabla }}_a {\tilde{\phi }} =0. \end{aligned}$$

In terms of the proper time $\tau$ along the fluid worldlines with tangent ${\tilde{u}}^a$, Eq. (55) reads

56 $$\begin{aligned}&{\tilde{u}}_a \left( \frac{d{\tilde{\rho }}^{(m)} }{d\tau }+{\tilde{\rho }}^{(m)} \, {\tilde{\nabla }}^c {\tilde{u}}_c \right) \nonumber \\&\quad +{\tilde{\rho }}^{(m)} \left( \frac{ d{\tilde{u}}_a}{d\tau } -\, \sqrt{ \frac{4\pi G}{2\omega +3}} \, {\tilde{\nabla }}_a\phi \right) =0, \end{aligned}$$

equivalent to

57 $$\begin{aligned} \frac{d{\tilde{\rho }}^{(m)} }{d\tau } + {\tilde{\rho }}^{(m)} \, {\tilde{\nabla }}^c {\tilde{u}}_c =0 \end{aligned}$$

and

58 $$\begin{aligned} \frac{D{\tilde{u}}^a }{D\tau } =\sqrt{ \frac{4\pi G}{2\omega +3}} \, \, {\tilde{\nabla }}^a {\tilde{\phi }}. \end{aligned}$$

The Einstein frame cousin of the geodesic equation is^3^ [20, 24, 25]

59 $$\begin{aligned} \frac{d^2 x^a}{d\tau ^2} +{\tilde{\varGamma }}_{bc}^a \, \frac{d x^b}{d\tau } \, \frac{d x^c}{d\tau } = \sqrt{ \frac{4\pi G}{2\omega + 3}} \, \, {\tilde{\nabla }}^a {\tilde{\phi }}. \end{aligned}$$

In general spacetimes, $\nabla ^c \phi$ does not point along the particle trajectory. However, in an unperturbed FLRW universe, $\phi =\phi (t)$ and $\nabla ^c \phi$ does point in the time direction of comoving observers. In this case, we have a force parallel to the worldline of a massive particle in the quasigeodesic equation

60 $$\begin{aligned} \frac{d^2 x^a}{d\tau ^2} +{\tilde{\varGamma }}_{bc}^a \, \frac{d x^b}{d\tau } \, \frac{d x^c}{d\tau } = \sqrt{ \frac{4\pi G}{2\omega + 3}} \, \frac{d{\tilde{\phi }}}{d\tau _c}\, \frac{d\tau _c}{d\tau } \, s^a, \end{aligned}$$

where we have identified ${\tilde{u}}^a$ with $s^a$ to keep with our notation of the previous sections. This parallel force in scalar–tensor FLRW cosmology can be seen as a variation of the particle mass along its trajectory.

### Cosmological particle creation and cosmic “antifriction”

Another implementation of parallel forces is intimately related to cosmological particle production. Particle creation due to quantum processes in the early universe is equivalent to negative bulk pressures [40, 41] and the idea that inflation could be driven by such a mechanism has been explored [43–46]. Self-interaction within dark matter can also give rise to negative bulk stresses and it was natural to investigate whether this mechanism can explain the present acceleration of the universe [42]. This mechanism causes a cosmic “antifriction” on the dark matter fluid, i.e., forces acting on fluid particles that are antiparallel to the spacetime trajectories of the latter [42].

## Summary and conclusions

A geodesic curve can be parametrized affinely or non-affinely. The general mathematical definition of a curve is that a curve is an equivalence class, where the equivalence relation is a change of parametrization, therefore affinely- and non-affinely-parametrized geodesics coincide. This definition does not take into account the fact that the proper time is a physically preferred parameter along the trajectory. The worldline of a particle subject to a parallel force may coincide, point by point, with a timelike geodesic however, in general, it cannot be affinely parametrized keeping the proper time as the parameter. A freely falling frame along the curve does not, in general, coincide with the comoving frame associated with the observer subject to a parallel force and following the same worldline; the time coordinates of these observers, i.e., their proper times $\tau$ and $\tau _c$, do not coincide.

FLRW cosmology is an exception: by setting a special initial condition (i.e., synchronizing the velocity along the quasi-geodesic with the Hubble flow), the proper time can be made to coincide with the affine parameter.

One can speculate on particular physical realizations of a force parallel to the timelike trajectory of a particle subject to it. A natural realization occurs when the particle mass changes along the trajectory (for example in rockets and light sails). Another realization occurs in scalar–tensor cosmology and string cosmology, where the dilaton field acts in a way similar to the Brans-Dicke gravitational scalar field. At present, it is not clear whether there are other physically meaningful ways of achieving such parallel forces.

## Data Availability

This manuscript has no associated data or the data will not be deposited. [Authors’ comment: This manuscript has no associated data due to its theoretical nature.]

## Appendix A

Begin from the non-affinely parametrized geodesic equation

A.1 $$\begin{aligned} \frac{d^2 x^{\mu }}{d\lambda ^2} +\varGamma ^{\mu }_{\alpha \beta } \, \frac{d x^{\alpha }}{d\lambda }\, \frac{d x^{\beta }}{d\lambda }= \alpha (\lambda ) \frac{dx^{\mu }}{d\lambda }. \end{aligned}$$

A reparametrization $$\lambda \rightarrow \sigma (\lambda ) $$ produces

A.2 $$\begin{aligned} \frac{d x^{\mu }}{d\lambda }= & {} \frac{d x^{\mu }}{d\sigma } \, \frac{d \sigma }{d\lambda },\nonumber \\ \frac{d^2 x^{\mu }}{d\lambda ^2}= & {} \left( \frac{d\sigma }{d\lambda } \right) ^2 \frac{d^2 x^{\mu }}{d\sigma ^2} + \frac{d^2 \sigma }{d\lambda ^2}\, \frac{dx^{\mu }}{d\sigma } \end{aligned}$$

and changes Eq. (A.1) into

A.3 $$\begin{aligned}&\left( \frac{d\sigma }{d\lambda } \right) ^2 \frac{d^2 x^{\mu }}{d\sigma ^2} + \frac{d^2 \sigma }{d\lambda ^2}\, \frac{dx^{\mu }}{d\sigma } + \left( \frac{d\sigma }{d\lambda } \right) ^2 \varGamma ^{\mu }_{\alpha \beta } \, \frac{dx^{\alpha }}{d\sigma } \, \frac{dx^{\beta }}{d\sigma } \nonumber \\&\quad = \alpha (\lambda ) \, \frac{d\sigma }{d\lambda } \, \frac{dx^{\mu }}{d\sigma }. \end{aligned}$$

The affine parametrization is obtained by imposing that

A.4 $$\begin{aligned} \frac{d^2\sigma }{d\lambda ^2} \, \frac{dx^{\mu }}{d\lambda } =\alpha (\lambda ) \, \frac{d\sigma }{d\lambda } \, \frac{dx^{\mu }}{d\lambda } , \end{aligned}$$

which leads to the second order ordinary differential equation

A.5 $$\begin{aligned} \frac{d^2\sigma }{d\lambda ^2} -\alpha (\lambda ) \frac{d\sigma }{d\lambda } =0 \end{aligned}$$

for the unknown function $\sigma (\lambda )$. This equation always admits the solution

A.6 $$\begin{aligned} \sigma (\lambda ) = A \int d \lambda \, \text{ e}^{ -\int d\lambda \, \alpha (\lambda )} +B, \end{aligned}$$

where *A* and *B* are integration constants.

## Footnotes

1 Other familiar coordinates in black hole spacetimes are based on radial *null* geodesics, including the Kruskal–Szekeres coordinates [26, 27].

2 Analytical solutions of the Einstein equations describing photon rockets have a long history [28–36].

3 A similar correction to the geodesic equation appears in dilaton gravity that results from the low-energy limit of string theories, but there the coupling of the dilaton may not be universal [21–23].
